# Multicentric reticulohistiocytosis (MRH): case report with review of literature between 1991 and 2014 with in depth analysis of various treatment regimens and outcomes

**DOI:** 10.1186/s40064-016-1874-5

**Published:** 2016-02-25

**Authors:** Saad Tariq, Steven T. Hugenberg, Stefanie A. Hirano-Ali, Hassan Tariq

**Affiliations:** Division of Rheumatology, Department of Medicine, Indiana University School of Medicine, 1120 West Michigan Street, Room CL 370, Indianapolis, IN 46202 USA; Dermatopathology Division, Department of Pathology, Indiana School of Medicine, Indianapolis, IN USA; Department of Histopathology, AFIP (Armed Forces Institute of Pathology) , Rawalpindi, Pakistan

**Keywords:** Arthritis, Autoimmune disease, Skin disease, Immunosuppressive medications

## Abstract

Multicentric reticulohistiocytosis is a rare disease affecting skin and joints primarily and rarely other organs. We present a case report of this disease and an extensive review of the literature. We reviewed the data between 1991 and 2014 and extracted 52 individual cases. Only articles in English were chosen after checking for relevance. The articles were studies and data was extracted into excel spread sheets and later used to compute such variables like frequency, mean and percentage of distribution of various clinical manifestations. The treatments used in these articles were critically analyzed and graded for their relative efficacy for skin and joint manifestations. The grades were 0 = worse, 1 = no benefit/condition remained same, 2 = improvement without resolution, and 3 = resolution. This article also reports the demographic, clinical, laboratory and pathological data from the reviewed articles. Authors attempted to discuss the findings of this review in depth to help manage this condition and proposed a treatment algorithm to help clinicians approach this rare and challenging disease.

## Background

Multicentric reticulohistiocytosis (MRH) is a rare systemic disease that can produce skin changes (usually papulo-nodular eruption), mucosal lesions, and arthritis, generally with erosive. It can rarely affect internal organs such as the lungs (resulting in pleural effusion) and heart (case reports of pericardial effusion and congestive heart failure), in addition to rare cases of mesenteric lymphadenopathy and urogenital lesions (Islam et al. 2013).

MRH is classified as non-Langerhans cell histiocytosis-class II b, per the Histiocyte Society recommendations (Zelger et al. 1996). It was first identified as a separate disease entity by Weber and Freudenthal (1937) with the term being coined by Goltz and Laymon (1954). Barrow et al. (1969) provided the first working definition of MRH.

At this time, there is not published data on incidence and prevalence of MRH. It has only largely been reported in single case studies. There are approximately 300 cases of MRH reported (Lesher and Allen 1984), mostly in Western countries and Japan. However, MRH is likely a world-wide disease with increased awareness and reporting in these certain regions. A recent article quotes MRH to more commonly affect females (2–3:1) (Tajirian et al. 2006) with mean presentation between 40 and 50 years of age (Luz et al. 2001).

Early and accurate diagnosis of MRH is crucial. When untreated, MRH can cause erosive arthritis with potential progression to arthritis mutilans. Skin manifestations may be the presenting, and sometimes only, sign of the disease. Biopsies from both skin and synovium may demonstrate numerous histiocytes with multinucleated giant cells and ground-glass appearing eosinophilic cytoplasm. The histiocytes contain periodic acid-Schiff (PAS) positive material.

We present a case of MRH and share our experience with treatment regimens. Additionally, we present a comprehensive review of the literature with attention to frequency of clinical manifestations, comorbid conditions, treatments, and treatment outcomes/benefits. Based upon this data, we have developed a suggested treatment algorithm.

## Case report

**Fig. 1:**
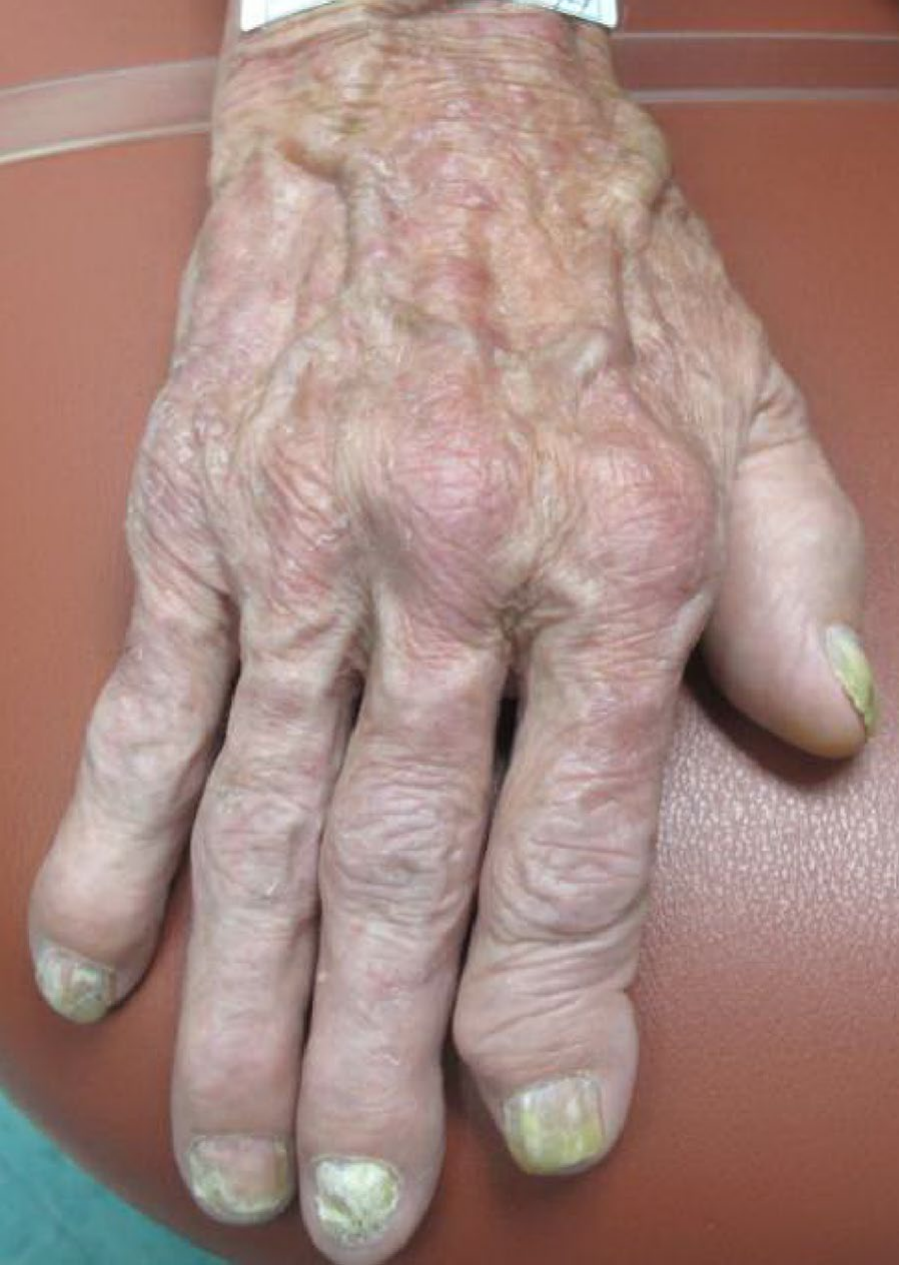
The patient’s right dorsal hand with visible swelling of all the distal, proximal interphalangeal and metacarpophalangeal joints

**Fig. 2:**
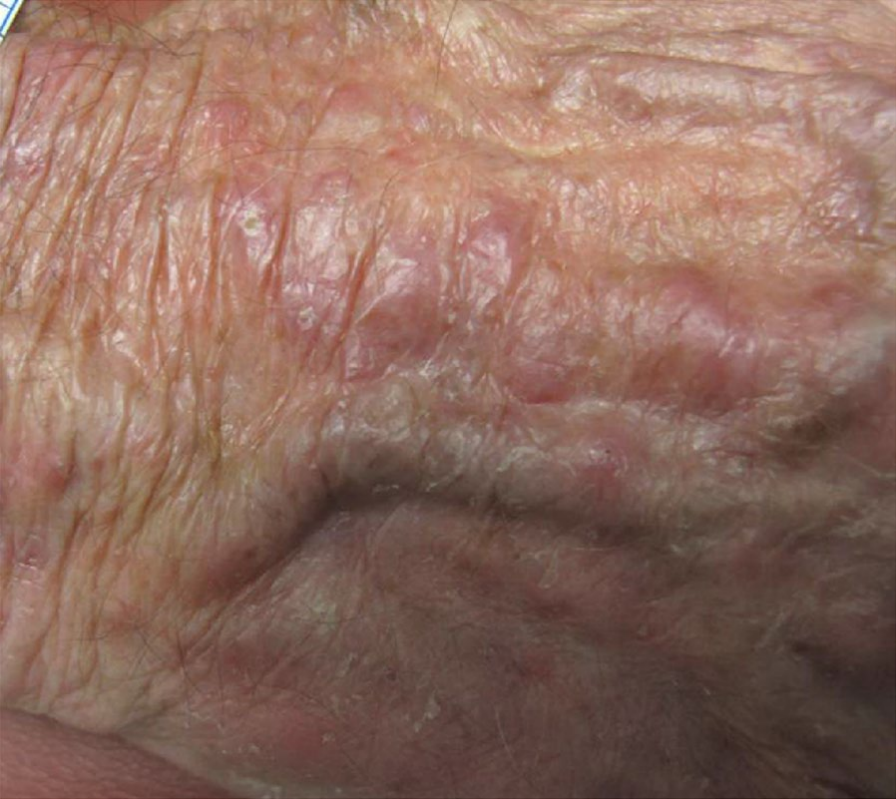
The right dorsal hand with multiple *red-brown* macules and papules

We present a case of a healthy 63-year-old Caucasian man who had simultaneous onset of skin lesions, joint pain, and morning stiffness of up to 2 h duration. He also reported a subjective fever, weight loss (10 lbs.), and weakness. On clinical exam, the patient had red-brown macules and papules symmetrically distributed on the dorsum of hands, elbows, periocularly, and periauricularly (Figs. 1, 2). Yellow translucent firm papules were noted on the helix of the left ear, glabella, and bilateral eyebrows. There was no lymphadenopathy. Joint examination revealed swelling and tenderness in proximal and distal interphalangeal joints (PIPs, DIPs), metacarpophalangeal joints (MCPs), elbows, shoulders, and knees.

An aspirate from his knee effusion revealed a total nucleated count of 5540/mm^3^ with 16 % neutrophils, 54 % lymphocytes, and 30 % monocytes. Synovial fluid analysis did not reveal crystals and cultures were negative. Serum laboratory studies were unremarkable, including erythrocyte sedimentation rate (ESR), complete blood cell counts, liver and renal function tests. His antinuclear antibody (ANA) and rheumatoid factor were negative.

**Fig. 3:**
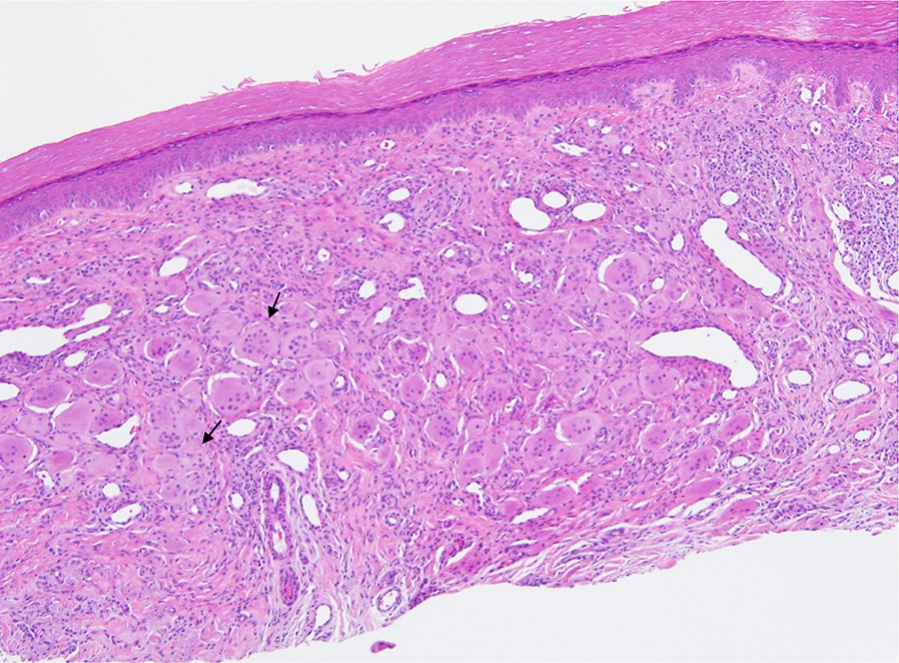
Skin biopsy from a *red-brown* papule on the right dorsal hand (Fig. 2) demonstrated numerous multinucleated histiocytes (*arrows*) infiltrating between the collagen bundles in the superficial dermis. There is a Grenz zone separating the epidermis from the dermal tumor. Additionally, there are an increased number of blood vessels amongst the histiocytes, as well as scattered lymphocytes

**Fig. 4:**
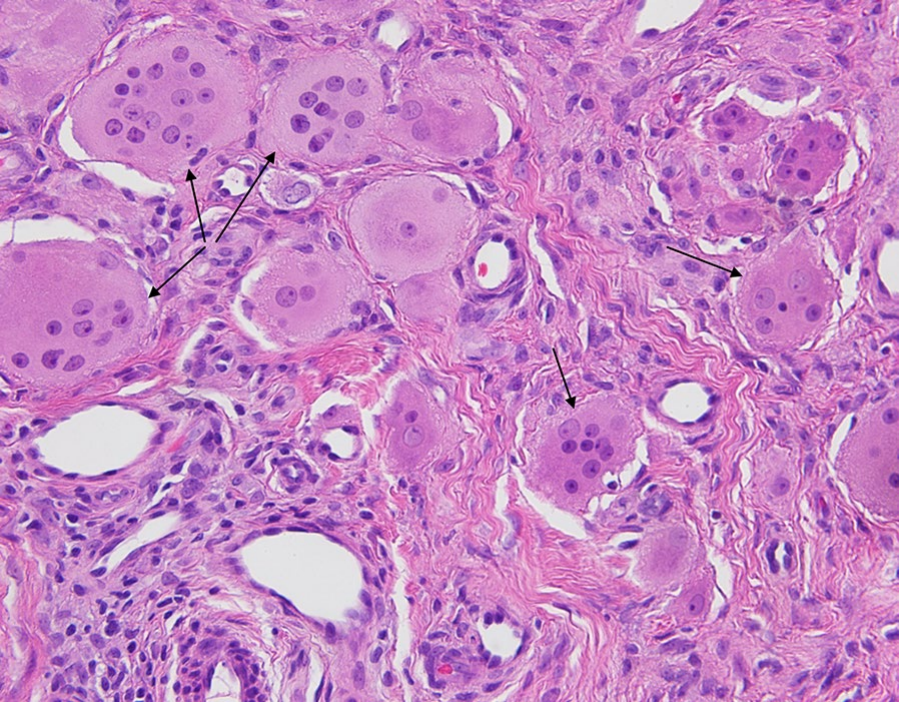
The multinucleated histiocytes (*arrows*) are large with an eosinophilic and finely granular “ground-glass” cytoplasm. The nuclei are haphazardly arranged, but tend to favor the center of the cells. A CD163 stain was diffusely positive and the cells were focally PAS-positive diastase-resistant

Initial clinical differential diagnosis included dermatomyositis, rheumatoid arthritis, and psoriatic arthritis. A skin biopsy demonstrated numerous multinucleated histiocytes infiltrating between the collagen bundles in the superficial dermis (Fig. 3). There is a Grenz zone separating the epidermis from the dermal tumor. The multinucleated histiocytes are large with an eosinophilic and finely granular “ground-glass” cytoplasm. The nuclei are haphazardly arranged, but tend to favor the center of the cells (Fig. 4). Additionally, there are an increased number of blood vessels amongst the histiocytes, as well as scattered lymphocytes. A CD163 stain was diffusely positive and the cells were focally PAS-positive diastase-resistant. The cells were negative for S100. Polarization did not reveal and polarizable material. These findings are diagnostic of MRH.

**Fig. 5:**
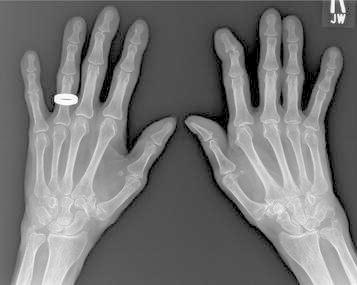
Radiographs of the hands showed erosions in the left third DIP and right second DIP, mild joint space narrowing with mild diffuse osteopenia, and erosions of the scaphoid bone with surrounding soft tissue swelling

**Fig. 6:**
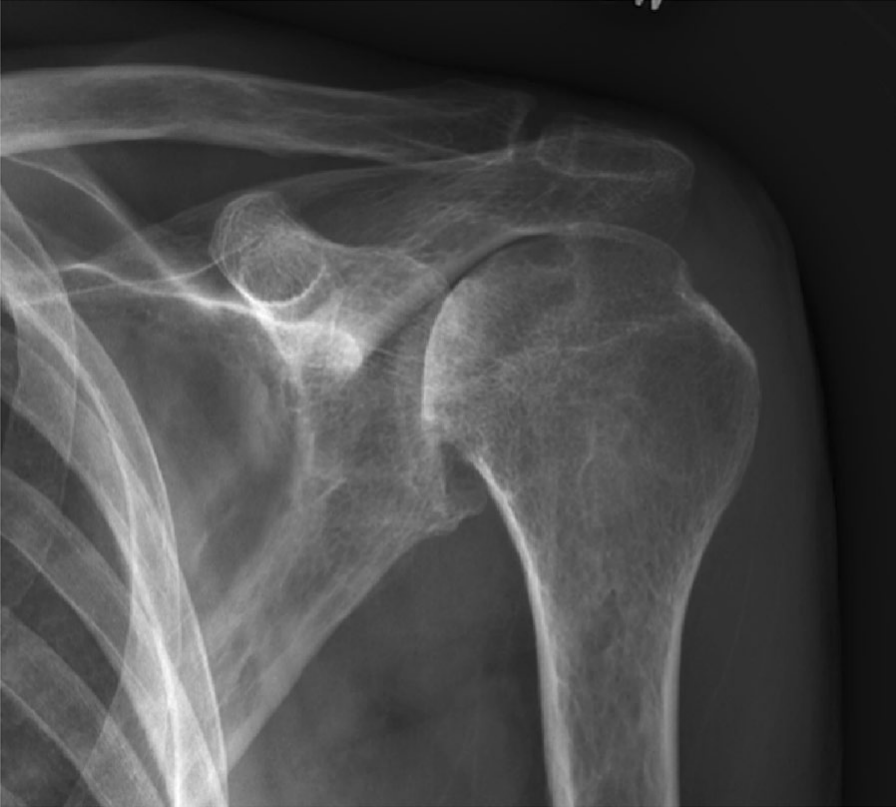
Radiograph of the left shoulder revealing marked glenohumeral joint space narrowing, small humeral head osteophytes, subchondral sclerosis, and subchondral cysts

**Fig. 7:**
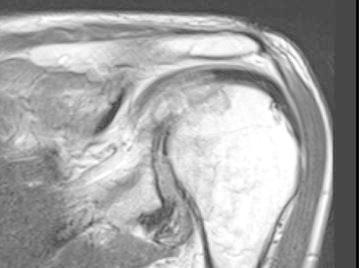
MRI of the shoulder showing moderate glenohumeral joint effusion with synovitis and large well-demarcated erosions on the humeral head

**Fig. 8:**
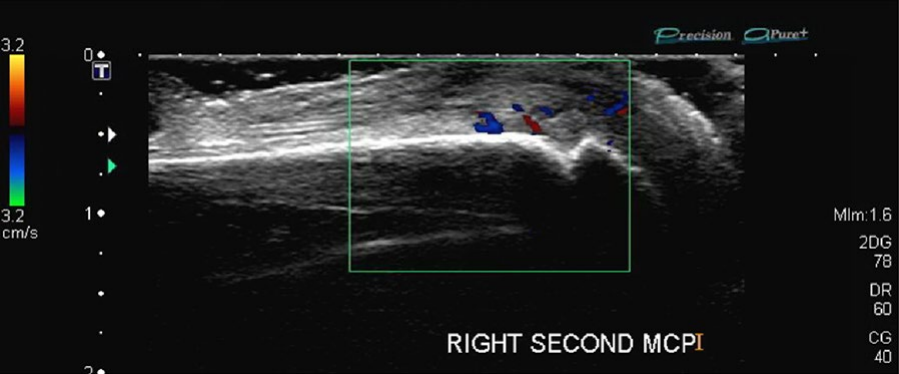
*Color* Doppler imaging demonstrating a minimal effusion but marked synovial proliferation with moderate Doppler flow

**Fig. 9:**
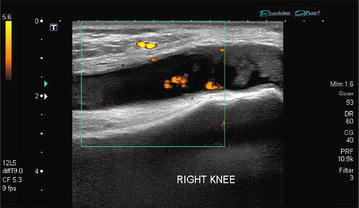
Large anechoic effusion and echogenic synovial proliferation with hypervascularity

Radiographs of the hands showed erosions in the left third DIP and right second DIP, in addition to mild joint space narrowing with mild diffuse osteopenia. There were also erosions seen in the scaphoid bone with surrounding soft tissue swelling (Fig. 5). Given the age of the patient concomitant osteoarthritis is the most likely cause of joint space narrowing. The patient’s left shoulder X-ray showed marked glenohumeral joint space narrowing, small humeral head osteophytes, subchondral sclerosis, subchondral cysts (Fig. 6), and large erosions of the humeral head. The bones were diffusely demineralized. Magnetic resonance imaging (MRI) of the shoulder showed moderate glenohumeral joint effusion with synovitis and well-demarcated erosions of the humeral head (Fig. 7). We performed an ultrasound on the second right MCP (Fig. 8) and first Interphalangeal joint which showed minimal effusion but marked synovial proliferation with moderate Doppler flow, suggesting disease activity. An ultrasound of the knee showed a large anechoic effusion, echogenic synovial proliferation with grade 2 Doppler flow (Fig. 9).

Given the association of MRH with internal malignancy (Trotta et al. 2004), the patient underwent cancer screening. This screening included colonoscopy and chest/abdominal computed tomography (CT). Purified protein derivative (PPD) skin test was negative. He was initially started on prednisone and methotrexate 20 mg/week with subsequent improvement in the appearance of his cutaneous disease. Due to continued pain and joint swelling after 3 months of treatment, he was started on adalimumab 40 mg subcutaneously every other week which was eventually increased to weekly dosing. Alendronate (70 mg weekly) was also started because of evidence in the literature of benefit in patients with MRH.

Over a follow-up period of 4 years the patient’s disease symptoms were largely controlled on the above medication regimen. However, imaging continued to show effusions in the hand joints and marked synovial proliferation in multiple MCPs, PIPs, and DIPs. Also, despite most of his symptoms improving, he had continued prominent right shoulder pain, with progressive disease shown on MRI, requiring joint replacement. He continued the use of the above medication regimen, and was able to taper his prednisone to 3 mg daily. Throughout his course, he continued to have radiologic evidence of disease despite relatively good symptom control.

## Methods

We performed a Pubmed search using the key words “multicentric reticulohistiocytosis” and limiting the results to those published between the years 1991–2014, yielding 227 articles. These articles were then individually screened for inclusion criteria including a diagnosis of MRH, written in the English language, and discussed treatment regimen and outcome. We reviewed the treatment options available (Islam et al. 2013; Zelger et al. 1996; Weber and Freudenthal 1937; Goltz and Laymon 1954; Barrow and Holubar 1969; Lesher and Allen 1984; Tajirian et al. 2006; Luz et al. 2001; Trotta et al. 2004; Havill et al. 1999; Lonsdale-Eccles et al. 2009; Muñoz-Santos et al. 2007; Goto et al. 2003; Bennàssar et al. 2011; Iwata et al. 2012; Hiramanek et al. 2002; Sakamoto et al. 2002; Cox et al. 2001; Flaming and Weigand 1993; Olson et al. 2015; Aouba et al. 2015; Eagle et al. 1995; Han et al. 2012; Teo and Goh 2009; Kishikawa et al. 2007; Valencia et al. 1998; Shiokawa et al. 1991; Moreau et al. 1992; Lambert and Nuki 1992; Qureshi et al. 1993; Gibson et al. 1995; Franck et al. 1995; Liang and Granston 1996; Kocanaogullari et al. 1996; Gorman et al. 2000; Morris-Jones et al. 2000; Hsu et al. 2001; Saito et al. 2001; Santilli et al. 2002; Blanco et al. 2002; Outland et al. 2002; Hsiung et al. 2003; Matejicka et al. 2003; Liu and Fang 2004; Kovach et al. 2004; Shannon et al. 2005; Mavragani et al. 2005; Malik et al. 2005; Gajic-Veljic et al. 2006; Adamopoulos et al. 2006; Chen et al. 2006; Chauhan et al. 2007; de Zwart-Storm et al. 2007; Codriansky et al. 2008; Satoh et al. 2008; Kalajian and Callen 2008; Broadwell et al. 2010; Kaul et al. 2010; Fett and Liu 2011; Mun et al. 2012; Yeter and Arkfeld 2013) and have summarized them in Table 1.

**Table 1:** Summary of the different treatments used with relative benefit

Medical treatments and indication	n total	n (%) for score 3 = resolution of the condition	n (%) for score 2 = improvement without resolution	n (%) for score 1 = no benefit/condition remained same	n (%) for score 0 = worse than prior to treatment
Methotrexate for arthritis	25	7 (28)	11 (44)	3 (12)	4 (16)
Methotrexate for skin	26	10 (38)	10 (28)	3 (11)	3 (11)
Hydroxychloroquine arthritis	7	0 (0)	1 (14)	3 (43)	3 (43)
Hydroxychloroquine skin	7	0 (0)	1 (14)	3 (43)	3 (43)
Thalidomide	1		1 (100)		
Sulfasalazine arthritis	1				1 (100)
Sulfasalazine skin	1				1 (100)
Leflunomide arthritis	2	2 (100)			
Leflunomide skin	2	2 (100)			
Azathioprine arthritis	2	1 (50)		1 (50)	
Azathioprine skin	2	1 (50)	1 (50)		
Cyclophosphamide arthritis	10	2 (20)	4 (40)	4 (40)	
Cyclophosphamide skin	11	3 (27)	5 (45)	3 (27)	
Cyclosporine arthritis	1	1 (100)			
Cyclosporine skin	2	1 (50)		1 (50)	
Etanercept arthritis	6	3 (50)	2 (33)	1 (17)	
Etanercept skin	6	3 (50)	2 (33)	1 (17)	
Adalimumab arthritis	2	2 (100)			
Adalimumab skin	2	2 (100)			
Infliximab arthritis	3	1 (33)	2 (67)		
Infliximab skin	3	1 (33)	2 (67)		
Alendronate arthritis	4	1 (25)	2 (50)	1 (25)	
Alendronate skin	4	2 (50)	1 (25)	1 (25)	
Zolendronic acid arthritis	3	1 (33)	2 (67)		
Zolendronic acid skin	2	1 (50)	1 (50)		
Pamidronate arthritis	1		1 (100)		
Pamidronate skin	1			1 (100)	
Chlorambucil arthritis	1				1 (100)
Chlorambucil skin	1				1 (100)
Sodium aurothiomalate	1			1 (100)	
Naproxen	1	1 (100)			
Prednisone alone for arthritis	2		2 (100)		
Prednisone alone for skin	2	1 (50)	1 (50)		

Data not available for blank boxes

n = total number of patients

The articles were then reviewed in depth with data collected including patient demographics, cutaneous and extra cutaneous manifestations, comorbid conditions, histological biopsy findings, laboratory studies, treatment regimens, and treatment outcomes. We graded the treatment response for each medication used with special attention to skin and joint disease. The following system was used to indicate the treatment responses; 0 = worse, 1 = no benefit/condition remained same, 2 = improvement without resolution, and 3 = resolution.

## Results

### Patient demographics

*Race and ethnicity* Thirty-nine of the 52 cases reported race/ethnicity. Fifteen cases (29 %) noted ethnicity to be “White,” 13 cases (25 %) reported origin from the “Far East,” 6 cases (11.5 %) reported race as “Black,” 3 cases (5.7 %) reported race as “Hispanic,” 1 case reported “Indian” origin, and 1 case reported “Middle Eastern” origin.

*Authorship country affiliation* The majority of the case reports came from the United States (18/35 %). The other cases originated from Japan (9/17 %), the United Kingdom (4/7.7 %), Spain (3/5.7 %), China (2/3.8 %), France (2/3.8 %), Ireland (2/3.8 %), India (1/1.9 %), Australia (1/1.9 %), New Zealand (1/1.9 %), Italy (1/1.9 %), Turkey (1/1.9 %), the Netherlands (1/1.9 %), and Taiwan (1/1.9 %). In 5 cases it was unclear as to which country the authors were affiliated (Table 2).

**Table 2:** Regional distribution of MRH cases

Countries	n = 52
United States	18
Japan	9
China	2
United Kingdom	4
Spain	3
France	2
Ireland	2
India	1
New Zealand	1
Autralia	1
Italy	1
Turkey	1
Netherlands	1
Taiwan	1

**Fig. 10:**
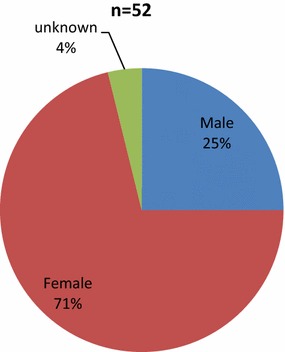
Gender distribution of the cases. Majority of the reported cases were female (71 %) while the rest were male. In few of the cases gender was not clearly reported

*Gender* Female cases predominated (37/71 %) above male cases (13/25 %), with a ratio of approximately 3:1 of women to men. In 4 % of cases the patient gender was not clearly noted (Fig. 10).

*Age* The mean age of reported disease onset was 46 years, while the mean age at diagnosis was 47.5 years. The youngest recorded case of disease onset was 8 years old from the Netherlands (Havill et al. 1999). The oldest recorded case of disease onset was 74 years old from the UK (Lonsdale-Eccles et al. 2009).

### Clinical features

Associated clinical features included cutaneous involvement in 50 cases (96 %), arthritis in 43 (82 %), weight loss in 10 (19 %), weakness in 8 (15 %), dysphagia in 5 (9.6 %), fatigue in 5 (9.6 %), fever in 5 (9.6 %), tenosynovitis in 3 (5.75 %), hoarseness in 3 (5.75 %), dry eye in 3 (5.75 %), myalgia in 2 (3.8 %), muscle atrophy in 2 (3.8 %), mucosal lesions in 2 (3.8 %), and pleural effusion in 2 (3.8 %) (Table 3). Additionally, nodules were noted in epiglottis in 3 cases and in the larynx in 2 cases (3.8 %). Other features noted (1 occurrence each) were pseudoptosis, macroglossia, upper respiratory infection symptoms, parotid enlargement, positive PPD, lymphadenopathy, Raynaud’s phenomenon, and splenomegaly. Concomitant medical conditions included Sjogren’s syndrome (3 cases), thyroid disease (2), hepatitis B (1), systemic lupus erythematosus (1), hypercholerstrolemia (1), cardiac failure (1), ulcerative colitis (1), primary biliary cirrhosis (1), and multiple sclerosis (1).

**Table 3:** Frequency of different clinical features in MRH reported

Clinical features	n = 52
Arthritis	43
Erosive disease	23
Arthralgia	5
Arthritis mutilans	4
Tenosynovitis	3
Skin lesions	50
Pseudoptosis	1
Weight loss	10
Weakness	8
Dysphagia	5
Fatigue	5
Fever	5
Nodules on epiglottis	3
Hoarseness	3
Sicca	3
Nodules in larynx	2
Muscle atrophy	2
Myalgia	2
Mucosal lesions	2
Macroglossia	1
Parotid enlargement	1
URI symptoms	1
PPD+	1
Lymphadenopathy	1
Raynaud’s phenomenon	1
Splenomegaly	1

Eleven cases (21 %) were associated with malignancy, but temporality with the diagnosis of MRH was not clearly stated. These malignancies included breast cancer (3), ovarian adenocarcinoma (2), ovarian neuroectodermal tumor (1), cutaneous squamous cell carcinoma (1), melanoma (1), papillary serous endometrial cancer (1), nasopharyngeal cancer (1), and hepatocellular carcinoma (1). Additionally, 2 patients had reported prior history of cancer, including cervical and colon origin.

#### Cutaneous involvement

**Fig. 11:**
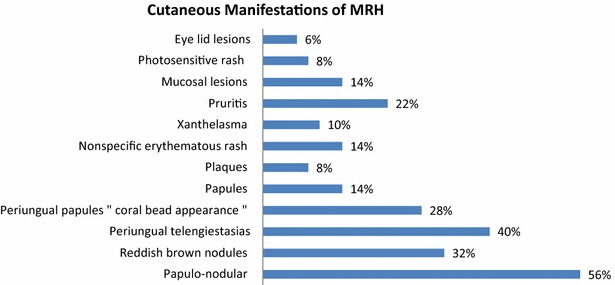
The most common skin manifestation of MRH was papulonodular rash followed by periungual telengiectasis. Classic coral bead appearance was seen in 28 % cases

Of the 50 cases with cutaneous involvement, the skin lesions were described as “papulonodular” 28 (56 %), “reddish brown” nodules 16 (32 %), “papular” 7 (14 %), “plaque-like” 4 (8 %), “macular” 2 (4 %), “erythematous rash” not otherwise specified 7 (14 %), “red confluent non-pruritic papules” 4 (8 %), and verrucoid 1 (2 %). Three (6 %) cases also had papules on the eyelids, described as firm, nontender, and mobile (Eagle et al. 1995) (Fig. 11).

Five cases documented a skin eruption with features similar to those seen in dermatomyositis. For example, one case reported pruritic and photodistributed erythema with telangiectasias on the trunk and neck (simulating the poikiloderma associated with dermatomyositis), in addition to erythematous-to-violaceous papules over the extensor surfaces of the fingers (simulating Gottron’s papules). This patient also had Raynaud’s phenomenon. However, only histopathology allowed the diagnosis of MRH (Muñoz-Santos et al. 2007).

Additional cutaneous findings included periungual papules with a coral bead appearance in 14 (28 %) cases, periungual telangiectasias in 2 (4 %), pruritus in 11 (22 %), oral mucosal nodules in 7 (14 %), xanthelasma in 5 (10 %), photodistributed erythema in 4 (8 %), bleeding tendency within the rash in 1 (2 %), tenderness in 1 (2 %) and neurofibroma-like nodules 1 (2 %) case.

#### Musculoskeletal involvement

**Fig. 12:**
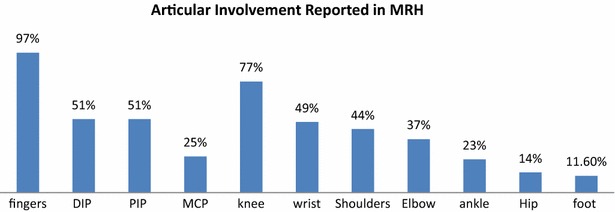
Hand involvement was the most common region with almost equal involvement of distal and proximal interphalangeal joints. Knee was the second most commonly involved joint with MRH

Findings of inflammatory arthritis from 43 cases showed finger involvement in 42 (97 %). DIPs were involved in 22 (51 %) cases, PIPs in 22 (51 %), MCPs in 12 (25 %), and carpometacarpal (CMC) in 4 (9 %) of cases (Fig. 12).

Other articular sited involved included the knees in 33 (77 %), wrists in 21 (49 %), shoulders in 19 (44 %), elbows in 16 (37 %), ankles in 10 (23 %), hips in 6 (14 %), joints of foot in 5 (11.6 %), and acromioclavicular joints in 2 (4.6 %).

Erosions were seen within the radiographs in 23 (55 %) cases and arthritis mutilans was reported in 4 (9.3 %) cases. Arthralgia alone was reported in 5 (11.6 %) cases while tenosynovitis was seen in 3 (7 %) cases. Periarticular osteopenia was seen in 3 (7 %), while flexion deformities from long standing disease were seen in 6 (14 %) of cases of arthritis.

### Laboratory findings

Laboratory values were reported in 38 of the 52 cases. Mean values included hemoglobin = 10.16 mg/dl, white blood cell count = 4581.66/mm^3^, ESR = 40.64 mm/h, C-reactive protein = 2.46 mg/dl, and creatinine kinase = 318 IU/ml. Rheumatoid factor was positive in 3 cases, anti-cyclic citrullinated peptide (CCP) was positive in 1 case, ANA was positive in 7 cases(the titer and dilution not recorded), anti-Ro was positive in 4, anti-La was positive in 1, anti-double-stranded DNA antibody was positive in 1, and perinuclear anti-neutrophil cytoplasmic antibodies (P-ANCA) were positive in 1 case. Low C-3 and CH50 were noted in 1 case each. A positive PPD was seen in only 1 case.

One case recorded the urine excretion of deoxypyridinoline in nM/mM as 19.3(normal range being 3.20 ± 0.75 (mean ± SD) nmol/mmol creatinine and 4.55 ± 1.22 nmol/mmol creatinine), N-telopeptide of type I collagen (NTX) in nM, bone collagen equivalents (BCE)/mM creatinine as 102.1 (normal range for men over 18 is 21–66 and for women is 19–63), bone alkaline phosphatase as 19.1 units/l, and osteocalcin as 6.6 ng/ml (Goto et al. 2003).

Bennassar et al., recorded elevated cytokine serum levels; tumor necrosis factor (TNF)-alpha level as 60 pg/ml (normal range 0–15 pg/ml), IL-6 as 525 pg/ml (0–5 pg/ml); IL-8 as 23 pg/ml (0–15 pg/ml); and IL-1β as 69 pg/ml (0–15 pg/ml) (Bennàssar et al. 2011).

Iwata et al., recorded the serum TNF-alpha level as 16.3 pg/ml and monocyte chemoattractant protein (MCP-1) level as 2790 pg/ml (normal 200–722 pg/ml) before treatment with infliximab 3 mg/kg. After the second infusion of infliximab the MCP-1 level decreased to 1350 pg/ml but the TNF-alpha level had increased (no values given) (Iwata et al. 2012).

### Histologic analysis

*Synovial biopsy* was done in 6 cases. Consistent findings included nodular infiltrate of plump histiocytes with abundant finely granular eosinophilic cytoplasm and multinucleated giant cells, and usually PAS positivity, diastase-resistant. Other finding noted cells with CD68 immunoreactivity, which failed to stain with S100 or CD1a (Hiramanek et al. 2002). One case mentioned that there was villous hypertrophic synovitis. Some degree of fibrosis was also noted in one case (Sakamoto et al. 2002).

*Skin biopsy* findings were recorded in 49 cases. The skin biopsy was done in vast majority of cases because of ease of access. The skin biopsies had similar histology to the synovial biopsies, in addition to absence of Birbeck granules on electron microscopy, positive staining for vimentin and lysozyme, prominent elastophagocytosis, and negative staining for Factor XIII-a, CD68 (KP-1), CD34, procollagen I, pan-actin, P75, CD45-RO, CD45-RA (Cox et al. 2001). Alcian blue stain failed to reveal interstitial mucin deposition in one case. Rare cases also noted positivity to alpha 1 anti-trypsin, and macrophage antigen compound (MAC)-387 stains (Cox et al. 2001). Additionally, CD1a and S100 stains were negative, indicating that the histiocytes are not Langerhans cells.

*Muscle biopsy* was done in 2 cases, one of which was normal and the other showed lipid laden uniform histiocytes with foamy cytoplasm in nodular aggregates (Flaming and Weigand 1993). Biopsy of *epiglottis nodules* in 1 case showed lipid laden uniform histiocytes (Flaming and Weigand 1993). Hypopharyngeal nodules were biopsied in 1 case showing findings consistent with MRH.

### Treatment comparison

The following medications were used with the number of instances in parentheses: prednisone (32), methotrexate (29), cyclophosphamide (12), nonsteroidal anti inflammatory drugs (NSAIDS) (11), bisphosphonates (9), hydroxychloroquine (8), etanercept (6), methylprednisolone (4), leflunomide (3), cyclosporine A (3), carboplatin (3), infliximab (3), azathioprine (2), adalimumab (2), intra-articular steroid injection (1), sulfasalazine (1), mycophenolate mofetil (1), chlorambucil (1), thiotepa (1), thalidomide (1), cisplatin (1), paclitaxel (1), sodium aurothiomalate (1), and minocycline (1). References (Islam et al. 2013; Zelger et al. 1996; Weber and Freudenthal 1937; Goltz and Laymon 1954; Barrow and Holubar 1969; Lesher and Allen 1984; Tajirian et al. 2006; Luz et al. 2001; Trotta et al. 2004; Havill et al. 1999; Lonsdale-Eccles et al. 2009; Muñoz-Santos et al. 2007; Goto et al. 2003; Bennàssar et al. 2011; Iwata et al. 2012; Hiramanek et al. 2002; Sakamoto et al. 2002; Cox et al. 2001; Flaming and Weigand 1993; Olson et al. 2015; Aouba et al. 2015; Eagle et al. 1995; Han et al. 2012; Teo and Goh 2009; Kishikawa et al. 2007; Valencia et al. 1998; Shiokawa et al. 1991; Moreau et al. 1992; Lambert and Nuki 1992; Qureshi et al. 1993; Gibson et al. 1995; Franck et al. 1995; Liang and Granston 1996; Kocanaogullari et al. 1996; Gorman et al. 2000; Morris-Jones et al. 2000; Hsu et al. 2001; Saito et al. 2001; Santilli et al. 2002; Blanco et al. 2002; Outland et al. 2002; Hsiung et al. 2003; Matejicka et al. 2003; Liu and Fang 2004; Kovach et al. 2004; Shannon et al. 2005; Mavragani et al. 2005; Malik et al. 2005; Gajic-Veljic et al. 2006; Adamopoulos et al. 2006; Chen et al. 2006; Chauhan et al. 2007; de Zwart-Storm et al. 2007; Codriansky et al. 2008; Satoh et al. 2008; Kalajian and Callen 2008; Broadwell et al. 2010; Kaul et al. 2010; Fett and Liu 2011; Mun et al. 2012; Yeter and Arkfeld 2013) and summarized in Table 1.

In cases using methotrexate for arthritis (25 cases), treatment resulted in a score of 3 (complete resolution of symptoms) in 28 % cases, a score of 2 (partial response) in 44 % of cases, a score of 1 (no benefit) in 12 % cases, and worsening of symptoms (score = 0) in 16 %. When treating for skin involvement (26 cases), treatment resulted in a score of 3 in 38 %, a score 2 in 28 %, a score of 1 in 11 %, and a score of 0 in 11 % of cases.

In case of cyclophosphamide use for the arthritis (10 cases), a treatment score of 3 was seen in 20 % cases, a score of 2 in 40 % of cases, and a score of 1 in 40 % cases. Use for skin lesions (11 cases), demonstrated treatment scores of 3 in 27 % cases, a score of 2 in 45 % cases, and a score of 1 in 27 % cases.

Hydroxychloroquine was used in 7 cases for both arthritis and skin lesions. No patients reached a treatment score of 3. A score of 2 was seen in 14 % of patients, a score of 1 in 43 % cases, and score of 0 in 43 % cases.

Azathioprine was used in 2 cases. One case had a score of 3 for skin and arthritis symptoms. The other case had a score of 2 for the skin symptoms and a score of 1 for the arthritis. *Leflunomide* was used in 2 cases and yielded score of 3 for skin and arthritis in both cases. Sulfasalazine use in 1 case resulted in a worse outcome (score 0) for skin and arthritis. Thalidomide use in 1 case resulted in partial response (score 2). Gold salt**s** (sodium aurothiomalate) were used in 1 case with no benefit (score 1). Similarly chlorambucil use was found to be ineffective, in fact worsening the disease in 1 case (score 0). Naproxen use in 1 case of pediatric disease onset resulted in complete resolution of symptoms. Although steroids were widely used, in 2 cases prednisone was used without any disease modifying anti-rheumatic drugs DMARD resulting in complete skin resolution in 1 case and partial response in the other. In both of these cases, the treatment for arthritis symptoms were partially beneficial without complete resolution (score 2).

Biological agents used included etanercept, adalimumab and infliximab. *Etanercept* was used in 6 cases giving complete response in 3, partial response in 2 and no benefit in 1 case. *Adalimumab* was used in 2 cases, resulting in complete response in both cases (score 3). *Infliximab* was used in 3 cases with complete response in 1 and partial response in 2 cases.

A recent case report mentions using Anakinra (IL-1 inhibition), which allowed control of the disease (score 3) (Aouba et al. 2015).

Bisphosphonates used included alendronate, zolendronic acid and pamidronate. Alendronate was used in 4 cases with scores for arthritis as follows: a score of 3 in 1 case, a score of 2 in 2 cases, and a score of 3 in 1 case. For skin lesions, treatment resulted in a score of 3 in 2 cases and a score 2 and 1 in 1 case each. Zolendronic acid use for arthritis resulted in treatment scores of 3 in 1 case and 2 in 2 cases. The use for skin symptoms resulted in a score of 3 in 1 case and a score of 2 in 1 case. Pamidronate use in 1 case resulted in score of 2 for arthritis, but only a score of 1 for skin symptoms.

*Combination drug use and second line therapies* In 18 cases methotrexate was initiated either after or concomitant with prednisone. This combination alone seemed to be successful in 10 cases (score of 3). Other medications were also combined with methotrexate, including 1 case with cyclophosphamide which controlled disease (score 3), 3 cases with hydroxychloroquine which did not provide sufficient disease control (score 2). The addition of cyclophosphamide or chlorambucil was also sub optimally effective and finally combination of methotrexate and etanercept controlled the disease. In two cases after using methotrexate alone, etanercept was added and was suboptimal (score 2), subsequent switching to infliximab was found effective in one case (score 3) and in the other leflunomide was combined with etanercept with an improved response (score 3). In 1 case each, using clodronate and zoledronic acid plus methotrexate was effective (score 3) after methotrexate alone was suboptimal (score 2). In 1 case of MRH associated with ovarian cancer, a combination of surgical excision of the cancer and chemotherapy lead to resolution of the MRH.

Prednisone alone was started in 6 patients with control of symptoms in 2 cases (3 score). In the rest, concomitant use of cyclophosphamide was successful (1 case with score of 3), methotrexate was effective in 3 cases (score 3), and cyclophosphamide plus methotrexate was useful in 1 case (score 3). In 1 case, use of prednisone with cyclosporine was of benefit (score 3) after suboptimal response with cyclophosphamide (score 2).

In one case with suboptimal benefit on the combination of etanercept and methotrexate (score of 2), use of adalimumab yielded better disease control (score 3).

In 4 cases with concomitant malignancy using chemotherapy and surgery for the primary cancer resulted in MRH remission as well (Han et al. 2012; Teo and Goh 2009; Kishikawa et al. 2007; Valencia et al. 1998).

## Discussion

Our results indicate that MRH is a worldwide disease with cases from around the globe and from various ethnic/racial backgrounds. More reports in White patients from Western countries may be attributed to higher awareness of the disease in these populations. MRH is easy to mistake for other inflammatory joint conditions such as rheumatoid arthritis or psoriatic arthritis, but the distinctive clinical, radiographic, and histologic features can aid in differentiating these diseases. Early disease may be difficult to diagnose prior to development of bone erosions. As the disease progresses, joint space narrowing of the distal interphalangeal DIPs, marginal erosions, and absence of new bone formation is a unique feature of MRH. Joint space narrowing and periarticular osteopenia may be present. Arthritis mutilans may be seen in untreated advanced disease (Islam et al. 2013). Although hands are affected most commonly, we found almost all the appendicular joints can be affected including wrists, elbows, shoulders, hips, knees, ankle, and the feet consistent with earlier reports (Barrow and Holubar 1969).

Our review found a significant female predominance with a ratio of 2.84:1 female to males, consistent with prior reports. The mean age of disease onset as 46 years, consistent with prior estimates, with the youngest case being an 8-year-old girl and the oldest age was 74 years. Since the conclusion of our review, a recent case report mentioned the occurrence of MRH in a 2-year-old girl (Olson et al. 2015).

Almost all the cases had either preceding or simultaneous cutaneous lesions which were variably described as “reddish brown nodules” or “papulo-nodular” in most cases. Other patterns described included “macular,” “papular,” and rarely “verrucoid.” A nonspecific “erythematous rash” was seen in few cases. In our case, and prior reports in the literature, the cutaneous eruption can mimic the findings seen in dermatomyositis such as findings that can be mistaken for poikiloderma, Gottron’s papules, and a heliotrope rash. Appropriate clinical, radiographic, and histologic correlation is necessary for accurate diagnosis of MRH.

At this time, if skin lesions are present, skin biopsy is the best test to diagnose MRH due to an absence of any unique biomarker for this disease. Generally there is negativity for rheumatoid factor, anti-CCP, ANA, and ANCA. ESR is typically elevated (mean of 40 mm/h), as is CRP (mean of 2.46 mg/dl.

Previously proposed treatment regimens have suggested use of NSAIDS in early and mild disease, or when treating the pediatric populations. However, given the general aggressive nature of this disease, early use of DMARD should be initiated once the diagnosis is made. Early use of steroids should be strongly considered, as there is evidence of benefit in this review with prednisone alone or in combination with other agents. Prednisone may be used initially in a moderate dose (7.5–≤30 mg/day) and then tapered to a lower dose (≤7.5 mg/day) based on clinical activity. Chronic low doses of prednisone may be required to control disease.

Our review shows that the most effective initial DMARD to use is methotrexate, which controlled arthritis symptoms (score 3) in 28 % cases and skin lesions in 38 %. Additionally, it showed partial disease control (score 2) in joints in 44 % and skin lesions in 28 % cases respectively. Other DMARDs such as leflunomide or azathioprine may be considered in cases with a contraindication to methotrexate. Other agents like hydroxychloroquine and sulfasalazine were not of significant benefit. Hydroxychloroquine may be considered as a combined agent.

Cyclophosphamide was found to be of significant benefit with 20 % of cases with complete (score 3) arthritis resolution and 27 % cases of skin lesions. Additionally partial arthritis and skin disease control (score 2) was seen in 40 and 45 % cases respectively. Today given the era of biologics, with relatively less systemic side effects and hassle of monitoring side effects, this powerful agent may be kept in reserve for refractory diseases or in cases of major extra-articular manifestations.

As new biologics are becoming available in rapid succession, it is prudent to incorporate these agents into our armamentarium to treat MRH. However, given the low number of case reports it is difficult to assess superiority of one biologic over another. Etanercept use was reported most often (6 cases) with clear disease control achieved in 3 cases and partial control in 2 cases. Adalimumab (2 cases) and infliximab (3 cases) were also shown to be effective. MRH is associated with elevations in TNF levels and therefore it seems logical to use anti-TNF agents to antagonize the disease pathway. It seems reasonable to combine these agents to help treat early aggressive disease with polyarticular or erosive disease with elevated inflammatory markers. Consideration should also be given to adding these agents when first line agents are unable to achieve any significant disease control in 4–6 weeks of follow up after initial visit. A recent case report mentions using anakinra (IL-1 inhibition), which allowed control of the disease (Aouba et al. 2015).

**Fig. 13:**
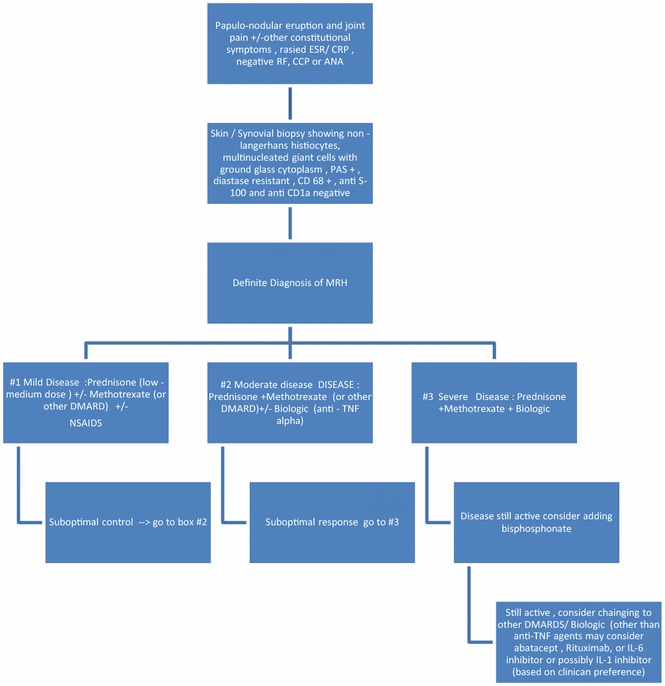
The algorithm summarizes the proposed treatment algorithm. For mild cases NSAIDs can be started but the disease is aggressive in most cases requiring steroids in varying doses and DMARDs. The Methotrexate can be used once the diagnosis is secured with biopsy. Given the erosive nature of the disease with possibility of joint mutilation and good response to biologic agents seen in multiple case reports especially the anti TNF agents, step up therapy should be considered in patients with suboptimal response to DMARDs

Bisphosphonates should be considered as “add-on” agent in cases of poor disease control or in cases of concomitant osteopenia (or osteoporosis) and/or steroid use. Our review suggests clear benefit from use of these agents. While it would be helpful to add these agents in the background of other DMARDs/biologics, their use alone would be hard to justify. Using zoledronate may be considered in a patient with continued disease activity while on DMARDs, biologics, and alendronate. Bone turnover markers, like urine NTX, may be checked before starting any bisphosphonate and then monitored to assure adequate efficacy. These markers may correlate with disease activity and may guide the choice of anti-resorptive agent. Elevated levels of bone turn over markers were shown at baseline in one case report, with a subsequent decrease after successful use of a bisphosphonate. The authors propose a treatment algorithm for MRH on the basis of literature review (Fig. 13).

Ultrasound can also be used to help guide treatment in MRH. In the current case being reported, using ultrasound we were able to see development of synovial proliferation and increased Doppler flow, both suggestive of continued disease activity. Additionally, ultrasound can help to objectively give information on erosions and soft tissue details of synovial and periarticular tissues.

The workup for a patient with MRH should always include age/gender appropriate cancer screening given the high association with malignancy. If concomitant malignancy is found, then treatment of the cancer may lead to resolution of the MRH (4 cases) (Eagle et al. 1995; Han et al. 2012; Teo and Goh 2009; Kishikawa et al. 2007). Additionally, the chemotherapy regimen in many cases, included cyclophosphamide, can also effective in treating MRH. Tuberculosis (TB) has an ill-defined association with MRH. Since we encountered only 1 case of latent TB, we cannot recommend routine screening. However, this may remain a wise exam to perform especially when using immunosuppressive agents to treat the disease. In our case, testing for TB was negative.

Given the association of MRH with osteopenia it is reasonable to consider a dual energy X-ray absorptiometry (DEXA) scan to establish a baseline level and to treat appropriately with the first sign of disease. Consideration for bisphosphonates should be given with high Fracture Risk Algorithm (FRAX) scores, osteopenia, or osteoporosis. Also, patients on chronic corticosteroids, even low dose, may receive benefit from this intervention added to calcium and vitamin D supplementation.

## Conclusion

MRH is a rare disease but with work wide prevalence. It is easily possible to confuse this with other more common autoimmune inflammatory conditions like rheumatoid arthritis or psoriatic arthritis. This disease has some unique clinical and radiographic features that set it apart from other inflammatory arthropathies. Prompt recognition and treatment of this disease is essential because if left untreated this can lead to progressive joint destruction and disability. In the absence of epidemiological studies or randomized control data, the authors hope that this systemic review of literature and the treatment algorithm will help the clinicians better identify and treat this condition (Fig. 13).
